# An Isolation Hospital's Ideas

**Published:** 1913-01-04

**Authors:** 


### AN ISOLATION HOSPITAL'S IDEAS.

Special interest attaches to the institution of the Conway and Penmaenmawr Joint Hospital Board, being, as it is, the first hospital in North Wales to receive patients under the King Edward National Memorial for Wales and also in connection with the Insurance Act. For the Board have placed twenty beds at the disposal of the Memorial Committee, and patients have been in residence for several weeks.

This new isolation hospital is situated at Groesynyd, in the beautiful Conway Valley, only two and a half miles from Conway itself.

Christmas morning was ushered in by the staff singing carols outside the wards, which had been brilliantly decorated by the united efforts of patients and nurses. An excellent Christmas dinner, with its usual accompaniments, was provided by the Board, whilst dessert together with a tea abounding in seasonable dainties were the gift of a neighbouring resident, Mr. G. Barker, and a few friends. During the afternoon songs and recitations were given in the various wards; in the evening games were indulged in.

